# Towards a cross-modal perspective of emotional perception in social anxiety: review and future directions

**DOI:** 10.3389/fnhum.2014.00322

**Published:** 2014-05-15

**Authors:** Virginie Peschard, Pierre Maurage, Pierre Philippot

**Affiliations:** Laboratory for Experimental Psychopathology, Institute of Psychology, Université Catholique de LouvainLouvain-la-Neuve, Belgium

**Keywords:** cross-modality, emotion, social anxiety, face, voice

## Abstract

The excessive fear of being negatively evaluated constitutes a central component of social anxiety (SA). Models posit that selective attention to threat and biased interpretations of ambiguous stimuli contribute to the maintenance of this psychopathology. There is strong support for the existence of processing biases but most of the available evidence comes from face research. Emotions are, however, not only conveyed through facial cues, but also through other channels, such as vocal and postural cues. These non-facial cues have yet received much less attention. We therefore plead for a cross-modal investigation of biases in SA. We argue that the inclusion of new modalities may be an efficient research tool to (1) address the specificity or generalizability of these biases; (2) offer an insight into the potential influence of SA on cross-modal processes; (3) operationalize emotional ambiguity by manipulating cross-modal emotional congruency; (4) inform the debate about the role of top-down and bottom-up factors in biasing attention; and (5) probe the cross-modal generalizability of cognitive training. Theoretical and clinical implications as well as potential fruitful avenues for research are discussed.

## Introduction

Influential models of social anxiety (SA) implicate cognitive biases as maintaining factors (Clark and Wells, 1995; Rapee and Heimberg, 1997). The existing evidence concerning biases in SA has largely relied on faces (for a review, Staugaard, 2010). Particularly, there is strong support for attentional biases (AB) towards facial stimuli among high socially anxious (HSA) individuals. While some studies indicated a facilitated attention to threatening faces (Mogg et al., 2004; Pishyar et al., 2004), others demonstrated difficulties in disengaging attention from these cues (Buckner et al., 2010; Schofield et al., 2012). Significant efforts have also been directed at understanding the effect of SA on the interpretation of faces, but have yielded mixed results, possibly due to methodological differences in dependent variables, stimuli and tasks. While several studies indicate that SA modulates the interpretation of emotional facial expressions (e.g., ratings of the emotional cost for interacting with the expressor: Schofield et al., 2007; Douilliez et al., 2012), other studies did not find any differences between HSA and controls (e.g., disapproval ratings: Douilliez and Philippot, 2003; decoding accuracy: Philippot and Douilliez, 2005).

To date, evidence linking SA to cognitive biases provided much information about how HSA individuals process faces. However, conclusions from these studies are limited to the processing of faces. Further, some questions are still controversial, in part due to the inherent methodological limitations of face research. Social interactions mobilize multiple channels, including speech style, facial expressions, postures, gestures, and tone of voice. Focusing research solely on faces raises the risk of overlooking other channels that are heavily implicated in social interactions. We argue that the investigation of SA-related biases needs to be extended to a multi-modal approach (as also suggested by Gilboa-Schechtman and Shachar-Lavie, 2013; Schulz et al., 2013), including the modalities that are most important in social interaction: vision and hearing. The use of cross-modal paradigms will allow the re-evaluation of studies using uni-modal stimuli, which could underestimate the cognitive biases present in real life. To support this statement, we developed several arguments based on empirical evidence, with the aim of identifying useful avenues for future research.

## Arguments

### Including emotional prosody to probe the generalizability of cognitive biases in social anxiety

Emotional prosody refers to all changes in acoustic parameters, such as intonation, amplitude, envelope, tempo, rhythm and voice quality during emotional episodes (Grandjean et al., 2006). It is a powerful communication tool transmitting paralinguistic information, and notably the speaker’s emotional state (Belin et al., 2004). Research that neglects the latter channel ignores crucial information for interpersonal interactions. To document its relevance, we will review research on the modulation of attention and emotional judgments by prosody.

#### Selective attention to emotional prosody

Efficient detection of salient or goal-relevant stimuli is essential to adjust behaviors accordingly. Given the limited processing capacity of our brain, mechanisms of attention play a critical role in selecting most important information from the myriad of sensory inputs. In the competition for processing resources, emotions have been shown to modulate attention (Vuilleumier et al., 2004; Vuilleumier, 2005). To date, the effect of emotional prosody on attention has been mostly assessed during dichotic listening or during the variation of feature-based attention.

The dichotic-listening technique is an attentional filtering task that assesses the ability to suppress or ignore distractors co-occurring with targets. Dichotic-listening investigations typically involve the simultaneous presentation of lateralized male and female voices with identical or different emotional prosody. Participants are requested to focus their attention on one ear and to determine the gender of the speaker on the attended ear. Recently, Aue et al. (2011) reported that, compared to neutral prosody, angry prosody attracts attention and induces behavioral and physiological changes (e.g., increased forehead temperature) with or without voluntary attention. Moreover, neuroimaging studies indicated greater activation for angry relative to neutral prosody in the superior temporal sulcus (Grandjean et al., 2005; Sander et al., 2005) and the amygdala (Sander et al., 2005) irrespective of the focus of attention. These findings suggest that threatening voices might be processed automatically by specific brain regions (but see Mothes-Lasch et al., 2011).

In addition to dichotic-listening methods, several studies (Quadflieg et al., 2008; Ethofer et al., 2009) investigated whether brain responses to angry compared to neutral prosody are modulated by variations in feature-based auditory attention. For example, Quadflieg et al. (2008) examined brain responses to neutral and angry voices while control and HSA subjects judged either the emotion or the gender of the voice. This study confirmed the findings of Sander et al. (2005) showing stronger activation for angry than neutral prosody in amygdala regardless of the task and in orbitofrontal cortex (OFC) during task-relevant as compared to task-irrelevant emotional prosody processing. Additionally, their results indicated that compared to controls, HSA individuals exhibit stronger right OFC response to angry versus neutral prosody regardless of the focus of attention. These findings suggest that the OFC might be implicated in biased processing of threatening prosody in SA.

To conclude, few studies have explored the implicit and explicit processing of emotional prosody via uni-modal attentional distraction from emotion. The lack of studies examining attention to prosodic information in the general population as well as in socially anxious samples is surprising, since the exploration of these processes could contribute to new insights into the attentional processing of emotional information. The above mentioned paradigms offer an interesting opportunity to provide evidence from the auditory modality that might be congruent or incongruent with the evidence accumulated in the visual domain.

#### Interpretation of emotional prosody

Other studies have focused on the interpretation of affective signals conveyed by faces or voices. These abilities have been increasingly studied in several psychopathologies, including alcohol-dependence (Maurage et al., 2009; Kornreich et al., 2013), depression (Naranjo et al., 2011) and bipolar disorder (Van Rheenen and Rossell, 2013).

Despite this growing interest, we found only one study (Quadflieg et al., 2007) that probed the presence of biases in the interpretation of emotional prosody in SA. Findings indicated that compared to controls, HSA participants present higher correct identification rates for fearful and sad prosody than controls, but conversely show impaired performances for happy prosody. Surprisingly, there were no differences between groups for neutral, anger and disgust prosody, as well as with regard to valence and arousal ratings for any prosody. These findings suggest that HSA individuals interpret prosody in a different manner than low socially anxious (LSA) individuals. However, it should be noted that this observation is at odds with theoretical predictions of a threat-specific bias, since fearful and sad expressions do not specifically indicate a social threat as would angry expressions do, thereby highlighting the importance of further investigations.

#### Summary

The lack of studies on emotional prosody in SA is problematic, since a threatening voice is a clear sign of danger and therefore a good candidate for capturing the attention of HSA individuals and eliciting biased interpretations. The study of emotional prosody constitutes a promising tool to investigate the cognitive biases in SA more completely. Presently, it is unclear whether these biases, which are repeatedly described in SA for visual processing, are similar in the auditory channel. Yet, the few existing data suggest some particularities in the processing of emotional prosody by HSA individuals. In addition to emotional prosody, other affective stimuli could be useful to probe the generalizability of cognitive biases in SA, notably body language (for an illustration in depression see Loi et al., 2013).

### Providing insights about the potential influence of social anxiety on the interactions between modalities

#### Audio-visual integration

A specific line of research addresses the ability of humans to integrate co-occurring sources of facial and vocal affective information. In natural environment, humans are immersed in a stream of stimulations from multiple modalities. The ability to integrate these multimodal inputs allows for an unified and coherent representation of the world and for taking advantage of non-redundant and complementary information from a single modality (Ernst and Bülthoff, 2004). The multimodal integration of affective facial and vocal expressions has led to a growing interest in the literature (for a review, Campanella and Belin, 2007). It has been demonstrated that congruency in the facial and vocal expression of emotion facilitates their identification compared to an uni-modal (i.e., face or voice presented in isolation) source of information (e.g., Collignon et al., 2008). Interestingly, integrative processes have been shown to be altered during the emotional perception of facial and vocal expressions in psychopathological populations, such as in alcohol-dependent subjects (Maurage et al., 2007, 2008, 2013). Specifically, alcohol-dependent individuals do not only suffer from a deficit in decoding facial and vocal expressions, but they also present a specific deficit in integrating messages conveyed by these two modalities. Hence, their resulting impairment is not just the sum of impairments in each modality, but it is further aggravated by a difficulty in integrating these modalities.

To our knowledge, no study has investigated the effect of SA on the ability to decode emotions in audio-visual modality, and the possible deficit in integrating these two modalities. This issue is important, as it would suggest that the total deficit in emotional information processing by HSA individuals would not be the addition of the deficits in each modality, but would be even more important, given the over-added integration deficit. Hence, the closer a paradigm would be to a real-life multi-sensory situation, the more pronounced might be the biases. Consequently, earlier uni-modal studies might have underestimated the extent of these biases.

#### Cross-modal attention

A second line of research has investigated how signals from different modalities influence each other in capturing attention. It has been shown that emotional prosody can serve as an exogenous cue to orient attention towards relevant visual events. Using a cross-modal adaptation of the dot-probe task, Brosch et al. (2008) showed decreased response times to non-emotional visual targets preceded by angry prosody compared to targets preceded by neutral prosody. Brosch et al. (2009) replicated and extended these behavioral findings by showing an amplification of the P1 (an electrophysiological component indexing early visual processing) for visual targets occurring at the spatial location of angry as compared to neutral prosody. These results suggest that emotional attention can operate across modalities because auditory stimuli can enhance early visual processing stages.

Several studies similarly demonstrated that emotional stimuli in one modality influence the processing of emotional information in another modality. For example, emotional prosody can facilitate attention to emotionally congruent facial expressions in visual search (Paulmann et al., 2012; Rigoulot and Pell, 2012) and in cross-modal priming tasks (Pell, 2005a,b; Paulmann and Pell, 2010). Other studies revealed that the judgment of emotional prosody is biased by a concurrent emotional face despite the instruction to ignore this channel (de Gelder and Vroomen, 2000; Vroomen et al., 2001). The reverse effect has also been observed, showing that emotional prosody biases the judgment of the emotion expressed in the face (de Gelder and Vroomen, 2000). These studies suggest that audio-visual integration of emotional signals may be an automatic and mandatory process, as this effect seems to arise independently of voluntary attentional factors (de Gelder and Vroomen, 2000; Vroomen et al., 2001) and of the awareness of the face (de Gelder et al., 2002).

Based on this line of research, one would want to investigate whether such automatic control of attention across modalities is modulated by SA. Such research could help identifying the origin of the SA biases on the top-down—bottom-up continuum. One could also hypothesize that HSA individuals could be more influenced than LSA individuals by cross-modal interference, if that interference can be interpreted as a social threat. These kind of studies need still to be conducted. The results obtained in healthy populations also raise the question of how conflicting emotional information is processed by HSA individuals. This topic will be developed in the next section.

### Manipulating the cross-modal emotional congruency as a tool to operationalize ambiguity

In the environment, we frequently encounter conflicting situations in which two modalities convey incongruent information (De Gelder and Bertelson, 2003). As mentioned, the categorization of emotional stimuli is affected by incongruent information provided by the second channel in cross-modal situations. Few studies have investigated such cross-modal incongruence effects among psychopathological populations. Some studies have described disturbed cross-modal integration of emotional faces and voices in schizophrenia (de Gelder et al., 2005; de Jong et al., 2009). However, no study has explored the effect of SA on the ability to decode incongruent emotional faces and voices. Yet, in real-life conditions, conversational partner often do not provide direct unambiguous feedback about their approval or disapproval. Such ambiguity leaves room for the socially anxious’ tendency to interpret responses as signs of negative evaluation. Recently, Koizumi et al. (2011) used a cross-modal bias paradigm (Bertelson and De Gelder, 2004) that included emotionally congruent or incongruent voice-face pairs. Participants had to decode the emotion displayed in one of the two channels (e.g., face) while ignoring the other (e.g., voice). Results indicate that individuals with heightened trait anxiety were likely to interpret the stimuli more negatively, putting more weight on the to-be-ignored angry faces or voices. As a consequence, manipulating emotional congruency across modalities can be a powerful way to examine the impact of ambiguity on the judgment of social information and to renew the exploration of biases in SA.

### Informing debate about the role of top-down and bottom-up factors in biasing attention to threat

Different models of anxiety have questioned the balance between bottom-up and top-down attention to explain cognitive biases. First, Bishop (2007) proposes that anxiety leads to AB by amplifying amygdala responsiveness to threat and/or by impairing the recruitment of top-down attention control, particularly under conditions of low perceptual load. In the same vein, the attentional control theory (Eysenck et al., 2007) and recent developments (e.g., Berggren and Derakshan, 2013; Berggren et al., 2013) suggest that individuals reporting high trait anxiety have to engage a greater amount of attentional control under low cognitive load (thereby reducing efficiency) to attain the level of performance achieved by low-anxious individuals. However, high cognitive load can disrupt performance in tasks requiring attentional control particularly in high anxious individuals. Finally, Hirsch and Mathews (2012) propose that high levels of anxiety are characterized by an imbalance between (weak) top/down and (strong) bottom/up attentional processes, the latter being automatically fueled by threat.

While behavioral studies demonstrated a rapid orientation towards threatening faces (Mogg et al., 2004; Pishyar et al., 2004), neuroimaging studies showed increased amygdala response, exaggerated negative emotion reactivity, and reduced cognitive regulation-related neural activation to faces in SA (Goldin et al., 2009; Ball et al., 2012). An increased vigilance for faces, indexed by enhanced P1, is also well documented in SA (Rossignol et al., 2012; Peschard et al., 2013). Nevertheless, most of this research is limited to visual stimuli and therefore prevents us from drawing firm conclusions about the implication of top-down and bottom-up factors in the generation of cognitive biases. Investigating the presence of biases across modalities offers an interesting paradigm to provide an insight into the contribution of top-down and bottom-up influences. Indeed, if a bias is generated at an early perceptual level, and thus nested in a specific modality, it is unlikely that the same bias would be reproduced in all other modalities. Consequently, the absence of generalization of a cognitive bias across modalities would support the notion that this bias is yielded by bottom-up processes, whereas its presence across modalities would rather support the notion of a top-down influence. As far as we know, no study has yet explored these integrative processes in SA, thus stressing the need to initiate this field of research.

### The cross-modal generalizability of cognitive training

Recent studies have shown that training HSA individuals to attend to non-threatening stimuli reduces AB, which in turn diminishes anxiety (Amir et al., 2008; Heeren et al., 2012b). It has also been demonstrated that inducing AB for threat induces anxiety (Heeren et al., 2012a). These findings support the proposal that AB to threat play a causal role in the maintenance and the development of SA. However, previous research has left unaddressed several important issues both at the fundamental and clinical level. First, there is a need to obtain a more ecological and complete AB evaluation before AB training. It should be established whether similar AB are present across modalities (as posed by theoretical models) or whether they are proper to a specific modality, hence suggesting retraining in that specific modality. Moreover if research findings show that AB appear across modalities, a crucial question would be whether training in one modality would transfer its effects to other modalities. This cross-modal perspective can offer an interesting paradigm to disentangle top-down and bottom-up determinations of AB. Finally, this perspective could lead to innovative AB training based on the combination of different modalities.

## Conclusion

We developed several arguments pleading for a cross-modal perspective in the investigation of biases in SA. In addition to the gain of a more complete and ecological picture of cognitive biases, a cross-modal perspective opens up new possibilities for understanding fundamental processes underlying biases in SA. This perspective might help to determine the stage of processing at which these biases occur. In this contribution, we mainly focused on auditory and visual modalities. However, signals from other modalities, like olfaction, could also influence information processing and should thus be considered in psychopathological research (Maurage et al., 2014). Recently, Adolph et al. (2013) have reported that HSA individuals might be particularly sensitive to chemosensory contextual social information during the processing of anxious facial expressions. This outlines the usefulness to exploring cross-modal processing in order to precisely describe cognitive biases in SA.

## Conflict of interest statement

All authors report no competing financial interests or potential conflicts of interest, and no financial relationships with commercial interests. The authors are funded by the Belgian Fund for Scientific Research (F.N.R.S., Belgium), but this fund did not exert any editorial direction or censorship on any part of this article.
